# Ring finger protein 166 potentiates RNA virus-induced interferon-β production *via* enhancing the ubiquitination of TRAF3 and TRAF6

**DOI:** 10.1038/srep14770

**Published:** 2015-10-12

**Authors:** Hai-Wei Chen, Yong-Kang Yang, Hao Xu, Wei-Wei Yang, Zhong-He Zhai, Dan-Ying Chen

**Affiliations:** 1 Key Laboratory of Cell Proliferation and Differentiation of The Ministry of Education, School of Life Sciences, Peking University, Beijing 100871, China

## Abstract

Host cells orchestrate the production of IFN-β upon detecting invading viral pathogens. Here, we report that Ring finger protein 166 (RNF166) potentiates RNA virus-triggered IFN-β production. Overexpression of RNF166 rather than its homologous proteins RNF114, RNF125, and RNF138, enhanced Sendai virus (SeV)-induced activation of the IFN-β promoter. Knockdown of endogenous RNF166, but not other RNFs, inhibited the IFN-β production induced by SeV and encephalomyocarditis virus. RNF166 interacted with TRAF3 and TRAF6. SeV-induced ubiquitination of TRAF3 and TRAF6 was suppressed when endogenous RNF166 rather than RNF114/138 was knocked down. These findings suggest that RNF166 positively regulates RNA virus-triggered IFN-β production by enhancing the ubiquitination of TRAF3 and TRAF6.

Innate immunity provides a robust first line of defense against invading pathogens. After detecting invading viruses, host cells initiate several signaling cascades to generate type I interferons (IFNs) such as IFN-β and IFN-α. Type I IFNs activate the JAK-STAT pathway, resulting in expression of hundreds of interferon-stimulated genes, which can target every stage of the viral life-cycle and protect host cells from invading viruses^1^.

Members of the RLR family, including retinoic acid inducible gene-I (RIG-I), melanoma differentiation-associated gene 5 (MDA5), and laboratory of genetics and physiology 2 (LGP2), are located in the cytoplasm to monitor viral RNA^2^. Upon viral infection, the helicase domain of RIG-I and MDA5 sense viral RNA that bears a 5′-triphosphate group that is lacking in host mRNA^3^,^4^. After binding viral RNA, RIG-I and MDA5 undergo conformational changes as well as modifications with K63-linked polyubiquitin chains by TRIM25 and REUL (also known as Riplet or RNF135)^5^,^6^,^7^,^8^. Ubiquitinated RIG-I and MDA5 interact with VISA (also named MAVS, Cardif or IPS-1) and this results in aggregation of the latter^9^,^10^,^11^,^12^. VISA polymers then recruit TRAFs such as TRAF3 and TRAF6 to promote the ubiquitination reaction which is critical for recruiting IKK and TBK1 to the VISA signaling complex^13^. IKK and TBK1 phosphorylate VISA, resulting in binding of VISA to the conserved, positively-charged surfaces of IRF3, thereby recruiting IRF3 for phosphorylation and activation^14^.

The identity of the cytoplasmic DNA sensor remained unresolved until researchers recently identified cyclic GMP-AMP synthase (cGAS) as a new viral DNA sensor^15^,^16^,^17^. Upon DNA viral infection, cGAS directly binds to DNA and releases its catalytic pocket to ATP and GTP for the generation of 2′3′-cGAMP^18^,^19^,^20^,^21^,^22^. cGAMP binds to and activates STING to assemble a punctate structure that contains TBK1. TBK1 then phosphorylates STING, and this is followed by the recruitment of IRF3 to STING for phosphorylation and activation^14^.

Ubiquitination plays a critical role in the RNA virus-induced innate immune response. As noted above, K63 ubiquitination of RIG-I triggered by TRIM25 and REUL is indispensable for its activation^5^,^6^,^7^,^8^, while Ring-finger protein 125 (RNF125) and c-Cbl catalyze the K48-linked ubiquitination of RIG-I and negatively regulate RIG-I-mediated antiviral activity^23^,^24^. Ubiquitin carboxyl-terminal hydrolase CYLD, a de-ubiquitination enzyme, physically interacts with RIG-I and removes its K63-linked polyubiquitin chains to attenuate antiviral activity^25^. VISA polymers can also recruit ubiquitin ligase family members, multiple TRAFs, through different TRAF-binding motifs to promote K63-linked ubiquitination, thereby recruiting NEMO to the VISA complex, which turns on TBK1 and IKK, resulting in the activation of IRF3 and NF-κB^13^. In addition, cIAP1/2 acts as a positive regulator by enhancing RNA virus-mediated K63-linked ubiquitination of TRAF3/6, while OTUB1/2 plays an opposite role *via* deubiquitinating TRAF3/6^26^,^27^.

In this report, we show that Ring-finger protein 166 (RNF166) potentiates RNA virus-induced IFN-β production *via* enhancing the ubiquitination of TRAF3 and TRAF6. These findings broaden our understanding of the mechanisms by which RLR signaling is positively regulated upon viral infection.

## Results

### RNF166 rather than its homologous proteins potentiates RNA virus-induced IFN-β production

**Figure 1:**
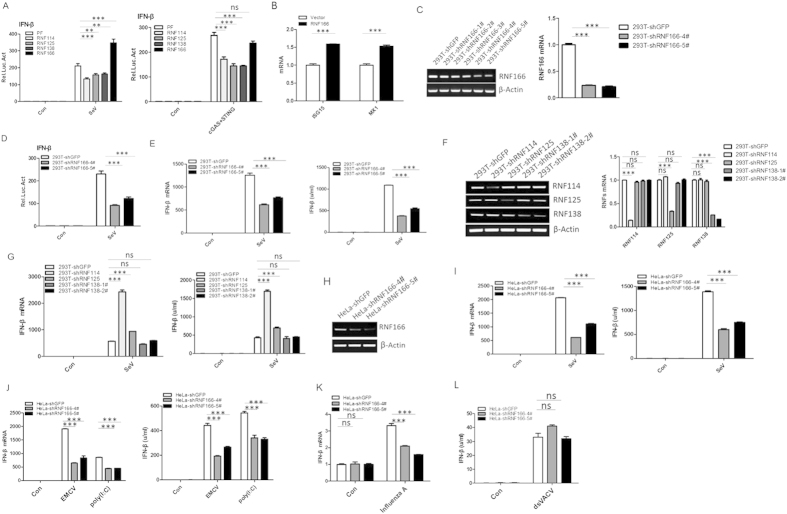
RNF166 rather than RNF114, RNF125, or RNF138 potentiates RNA virus-induced IFN-β production. (**A**) RNF166 positively regulates SeV- but not cGAS- and STING-induced activation of the IFN-β promoter. 293T cells (2 × 10^5^) were transfected with IFN-β reporter (50 ng), pRL-TK *Renilla* luciferase plasmid (50 ng), and the expression plasmids indicated above. 12 h after transfection, cells were infected with SeV or left uninfected, and luciferase assays were performed 24 h after infection. (**B**) RNF166 enhances the transcription of ISG15 and MX1. 293T cells (2 × 10^6^) were transfected with empty vector or expression plasmid of RNF166 (2 μg), 24 h after transfection, the mRNA of ISG15 and MX1 were detected by Q-PCR. (**C**) HEK293T cell pools with stable knockdown RNF166 were generated by shRNA. Knockdown efficiency was determined by RT-PCR (left) and Q-PCR (right). (**D**) SeV-mediated activation of the IFN-β reporter was inhibited when the endogenous RNF166 was stably knocked down. Transfection and luciferase assays were performed as in (**A**). (**E**) SeV-induced IFN-β production by 293T-shRNF166-#4 and #5 cells was lower than by 293T-shGFP cells. Cells (1 × 10^6^) were infected with SeV. 12 h after infection, cells were analyzed by Q-PCR (left) and the supernatants were collected for IFN-β bioassays (right). (**F**) HEK293T cell pools with stable knockdown of RNF114, RNF125, and RNF166 were generated using shRNA. Knockdown efficiency was determined by RT-PCR (left) and Q-PCR (right). (**G**) SeV-induced IFN-β production by cell pools with stable knockdown of RNF114, RNF125, and RNF138. Cells were infected and analyzed as in (**E**). (**H**) The knockdown efficiency of RNF166 in HeLa cells was determined by RT-PCR. (**I**–**L**) SeV-, EMCV-infection, poly (I:C)-transfection(4 ug) and Influenza A-infection, rather than dsVACV-transfection(4 ug) induced production of IFN-β by HeLa-shRNF166-#4 and #5 cells were lower than by HeLa-shGFP cells. Q-PCR and bioassays were performed at 12 h after treatment as in (**E**). Each graph represents the mean ± SD of three independent experiments done in triplicate. ***indicates P < 0.001; **indicates P < 0.01; ns (not significant) indicates P > 0.05.

RNF166 is closely related to RNF125, which has been reported to negatively regulate RIG-I- mediated anti-RNA virus signaling by conjugating ubiquitin chains to RIG-I and leading to the degradation of RIG-I by the proteasome^23^. RNF125 and its homologous proteins RNF114, RNF138, and RNF166 form a subfamily of small C3HC4 RING ubiquitin ligases^28^, so we investigated whether RNF114/138/166 also play a role in RNA virus-induced IFN-β production. We transfected plasmids that encoded RNF114, RNF125, RNF138, and RNF166 into HEK293T cells to perform reporter assays. We found that overexpression of RNF166 but not it’s homologous RNF114, 125, and 138, potentiated Sendai virus (SeV)-induced activation of the IFN-β promoter. However, RNF166 had no apparent effect on the overexpression of cGAS and the STING-induced activation of the IFN-β promoter (Fig. 1A), suggesting that RNF166 specifically enhances RNA but not DNA virus-induced IFN-β production. Overexpression of RNF166 can also enhance the transcription of Interferon-stimulated genes (ISGs) like ISG15 and MX1 (Fig. 1B).

To determine whether endogenous RNF166 is involved in anti-RNA virus signaling, we generated stable RNF166-knockdown cell pools using shRNA plasmids that targeted five sites on human RNF166 mRNA. Two shRNA plasmids (#4 and #5) markedly inhibited the expression of endogenous RNF166 mRNA in HEK293T cells, whereas the #1, #2, and #3 shRNA plasmids had little effect on RNF166 mRNA (Fig. 1C). We found that knockdown of RNF166 significantly inhibited the activation of the IFN-β promoter, transcription of IFN-β mRNA, and the secretion of IFN-β triggered by SeV infection (Fig. 1D,E). We also generated stable RNF114-, RNF125-, and RNF138-knockdown HEK293T cell pools to determine their functions (Fig. 1F). Consistent with a previous report that RNF125 acts as a negative regulator of the RIG-I-mediated signaling pathway^23^, knockdown of RNF125 slightly enhanced the SeV-elicited IFN-β production compared to the control cell pool, while knockdown of RNF114 clearly enhanced the SeV-induced IFN-β production, and knockdown of RNF138 had no appreciable effect (Fig. 1G).

We further generated stable RNF166-knockdown cell pools with HeLa cells and obtained similar results; the production of IFN-β induced by SeV, encephalomyocarditis virus (EMCV) and human influenza A virus infection, as well as poly (I:C)-transfection notably decreased when endogenous RNF166 expression was knocked down (Fig. 1H–K). However, knockdown of RNF166 had no effect on DNA analog-induced activation of IFN-β (Fig. 1L). These results suggested that RNF166 rather than its homologous proteins physiologically potentiates RNA virus-induced IFN-β production.

### RNF166 targets TRAF3 and TRAF6 to potentiate VISA-mediated antiviral signaling

**Figure 2:**
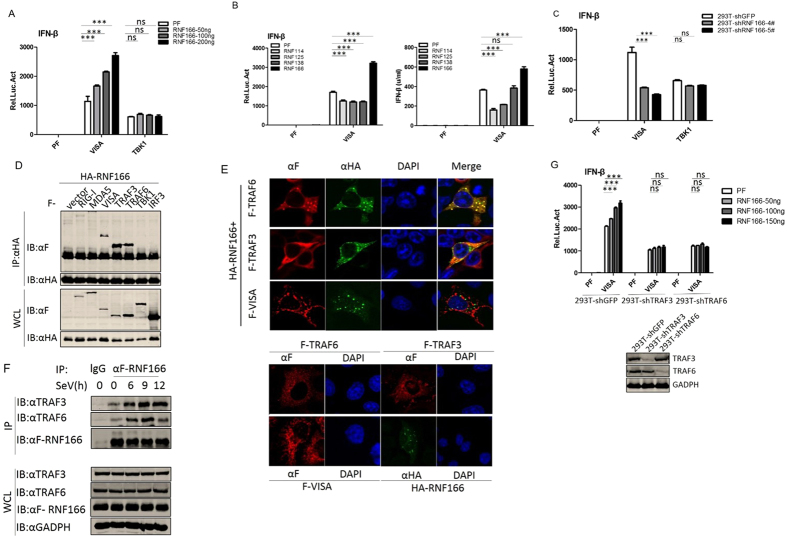
RNF166 targets TRAF3 and TRAF6 to potentiate VISA-mediated antiviral signaling. (**A**) RNF166 potentiates VISA- but not TBK1-mediated activation of the IFN-β promoter. 293T cells (2 × 10^5^) were transfected with IFN-β reporter (50 ng), pRL-TK *Renilla* luciferase plasmid (50 ng), and expression plasmids for VISA or TBK1 together with an empty vector and RNF166 construct (50, 100, and 200 ng). Luciferase assays were performed 24 h after transfection. (**B**) RNF166 rather than RNF114, RNF125, and RNF138 potentiates the VISA-mediated activation of the IFN-β promoter and production of IFN-β. 293T cells (2 × 10^5^) were transfected with the indicated plasmids. 24 h after transfection, the supernatants collected for IFN-β bioassays (right); Luciferase assays (left) were performed as in (**A**). (**C**) VISA- rather than TBK1-mediated activation of the IFN-β promoter was attenuated in 293T-shRNF166-#4 and #5 cells compared with 293T-shGFP cells. Luciferase assays were performed as in (**A**). (**D**) Overexpressed RNF166 interacts with overexpressed VISA, TRAF3, and TRAF6. 293T cells (1 × 10^7^) were transfected with the indicated plasmids (5 μg each). Cell lysates were immunoprecipitated (IP) with anti-HA (αHA). 24 h after transfection the immunoprecipitates and whole-cell lysates (WCL) were analyzed by western blot (IB) with anti-HA and anti-Flag antibody. (**E**) RNF166 co-localized with TRAF6 and TRAF3 but not VISA in HeLa cells. HeLa cells were transfected indicated expression plasmids (1 μg each). Immunofluorescent staining was performed with anti-Flag (red), anti-HA (green), and DAPI (blue). (**F**) Effects of SeV infection on the interaction between overexpressed RNF166 and endogenous TRAF3 and TRAF6. 293T cells (5 × 10^7^) were transfected with Flag-RNF166 plasmids (5 μg each). 18 h after transfection, cells were infected with SeV for the indicated times. The cell lysates were immunoprecipitated (IP) and analyzed by western blot (IB) with the indicated antibodies. (**G**) RNF166 did not potentiate the VISA-mediated activation of the IFN-β promoter in 293T-shTRAF3 and 293T-shTRAF6 cells. Luciferase assays were performed as in (**A**). Each graph represents the mean ± SD of three independent experiments done in triplicate. ***indicates P < 0.001; **indicates P < 0.01; ns (not significant) indicates P > 0.05.

We next determined which molecules are targets of RNF166 in the anti-RNA virus signaling pathway. In reporter assays, overexpression of RNF166 potentiated VISA-, but not the downstream kinase TBK1-mediated activation of the IFN-β promoter (Fig. 2A). Repoter Assay and Bioassay results showed that RNF166 but not the other RNFs potentiated VISA-mediated transcription and secretion of IFN-β, while the overexpression of RNF114 or RNF125 had inhibitory effects (Fig. 2B). Accordingly, knockdown of RNF166 inhibited VISA- but not TBK1-mediated activation of the IFN-β promoter (Fig. 2C). These data suggested that RNF166 acts on signaling components that are downstream of VISA and upstream of TBK1.

We next used co-immunoprecipitation to determine whether RNF166 interacts with VISA and its downstream components. Overexpressed RNF166 markedly associated with TRAF3 and TRAF6 in 293T cells, and also interacted with VISA, but had no detectable interactions with RIG-I, MDA5, TBK1, and IRF3 (Fig. 2D). RNF166 was subsequently shown to co-localize with TRAF3 and TRAF6 in co-transfected HeLa cells (Fig. 2E). Overexpressed RNF166 localized predominantly in the cytosol as dots which were probably aggregates of RNF166 protein. Double immunofluorescent staining showed that overexpressed TRAF6/3, especially TRAF6, had a similar distribution pattern and overlapped with RNF166, while we did not detect co-localization between RNF166 and VISA.

To define these interactions under physiological conditions, we set out to determine associations between endogenous RNF166 and the targets. As we could not obtain an effective antibody to detect endogenous RNF166 through preparation or commercial purchase, we performed immunoprecipitation between overexpressed RNF166 and endogenous TRAF3 and TRAF6 with or without SeV infection. Overexpressed RNF166 weakly interacted with endogenous TRAF3 and TRAF6, and SeV infection greatly enhanced these interactions (Fig. 2F). These data suggested that RNF166 associates with TRAF3 and TRAF6 during viral infection.

To further clarify whether RNF166 targets TRAF3 and TRAF6, we used shRNA to knockdown endogenous TRAF3 or TRAF6, and found that RNF166 no longer potentiated VISA-induced IFN-β activation when TRAF3 or TRAF6 expression was suppressed (Fig. 2G). These data further supported the idea that RNF166 targets TRAF3 and TRAF6 to potentiate RNA virus-induced IFN-β production.

### Both TRAF3 and TRAF6 play a critical role in SeV-induced interferon-β production in HEK293T cells

**Figure 3:**
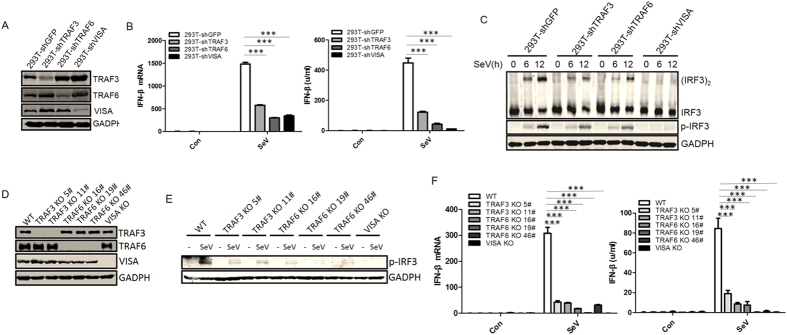
Both TRAF3 and TRAF6 play a critical role in SeV-induced signaling in HEK293T cells. (**A**) 293T cell pools with stable knockdown of TRAF3, TRAF6, and VISA were generated by shRNA. Knockdown efficiency was analyzed by western blot with anti-TRAF3, TRAF6, and VISA antibodies. (**B**) SeV-induced IFN-β production was reduced when endogenous TRAF3, TRAF6, or VISA was stably knocked down. Indicated cells (5 × 10^5^) were infected with SeV and IFN-β levels were estimated by Q-PCR (**B**, left) and bioassay (**B**, right) at the indicated times. (**C**) IRF3 dimer and p-IRF3 were attenuated in 293T-shTRAF3, 293T-shTRAF6, and 293T-shVISA cells infected with SeV. Indicated cells (5 × 10^5^) were infected with SeV for the indicated times and lysates were analyzed by western blot with anti-p-IRF3 and anti-GADPH or by native western blot with anti-IRF3. (**D**) TRAF3 and TRAF6 knockout HEK293T cell lines were generated by CRISPER/CAS9. Knockout cells were analyzed by western blot with anti-TRAF3, TRAF6, and VISA antibodies. (**E**) p-IRF3 was attenuated in TRAF3-knockout (KO) 293T, TRAF6-KO 293T, and VISA-KO 293T cells infected with SeV. Indicated knockout cells (5 × 10^5^) were infected with SeV and lysates were analyzed by western blot with anti-p-IRF3 antibody. (**F**) SeV-induced IFN-β was reduced when endogenous TRAF3, TRAF6, and VISA were knocked out. Q-RT-PCR (left) and IFN-β bioassay (right) were performed as in (**B**). Each graph represents the mean ± SD of three independent experiments done in triplicate. ***indicates P < 0.001; **indicates P < 0.01; ns (not significant) indicates P > 0.05.

That TRAF3 acts as a critical adaptor in RIG-I-mediated antiviral signaling has been demonstrated by several studies^29^,^30^, but its role has been doubted recently^13^. So we then determined whether TRAF3 or TRAF6 plays a critical role in SeV-induced IFN-β production by using shRNA to stably knock down endogenous TRAF3 or TRAF6 in HEK293T cells (Fig. 3A). We found that VISA-mediated activation of the IFN-β promoter, SeV-induced transcription, and the secretion of IFN-β were markedly reduced (Figs 2G and 3B), and SeV-induced phosphorylation and dimerization of IRF3 was apparently inhibited when TRAF3 or TRAF6 expression was suppressed (Fig. 3C).

To further determine the function of TRAF3 and TRAF6, we generated TRAF3 or TRAF6 knockout HEK293T cell lines using the CRISPR/Cas9 system (Fig. 3D). Phosphorylation of IRF3 triggered by SeV-infection was greatly inhibited and the production of IFN-β was blocked when TRAF3 or TRAF6 was deleted (Fig. 3E,F). These results suggested that both TRAF3 and TRAF6 play a critical role in SeV-induced IFN-β production in HEK293T cells.

### Functional domain mapping of RNF166

**Figure 4:**
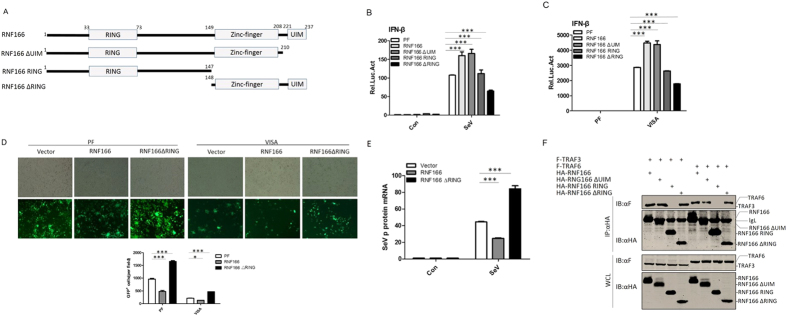
Functional domain mapping of RNF166. (**A**) Schematic structures of RNF166 and the deletion mutants used in this work. (**B**,**C**) Effects of truncations of RNF166 on SeV-induced (**B**) and VISA-mediated (**C**) activation of the IFN-β promoter. Transfection and luciferase assays were performed as above. (**D**) Effects of RNF166 and the RNF166 RING deletion mutant on VISA-mediated antiviral activity. 293T cells (5 × 10^5^) were transfected with the indicated plasmids (1 μg). At 20 h after transfection, cells were infected with NDV-eGFP at an MOI of 0.0001, and at 40 h after infection, viral replication was determined by fluorescence microscopy. The GFP-positive cells were counted and the percentage was calculated. (**E**) Effects of RNF166 and the RNF166 RING deletion mutant on replication of SeV. 293T cells (5 × 105) were transfected with the indicated plasmids (1 μg). At 12 h after transfection, cells were infected with SeV, and at 24 h after infection, mRNA of P protein was determined by Q-PCR. (**F**) RNF166 interacted with TRAF3 and TRAF6 *via* its zinc-finger domain. Transfection and immunoprecipitation (IP) were performed as in Fig. 2 (**D**). Each graph represents the mean ± SD of three independent experiments done in triplicate. ***indicates P < 0.001; **indicates P < 0.01; ns (not significant) indicates P > 0.05.

RNF166 contains a RING domain, a zinc finger domain, and an ubiquitin-binding domain (UIM) (Fig. 4A). To dissect the functional role of these domains, we generated three deletion mutants and assessed their ability to up-regulate SeV-induced IFN-β production. We found that the presence of an intact RING domain is essential for RNF166 function, as overexpression of a RING deletion mutant (RNF166 ΔRING) failed to enhance, but rather inhibited the activation of the IFN-β promoter induced by SeV and the overexpression of VISA, while the UIM domain deletion mutant (RNF166 ∆UIM) had no apparent effect (Fig. 4B,C). Consistently, on infection with NDV-eGFP (Newcastle disease virus-enhanced green fluorescent protein), we found that overexpression of RNF166 in HEK293T cells rendered them remarkably resistant to NDV infection and reduced the levels of NDV-eGFP-positive cells, while the RING deletion mutant had the opposite effect (Fig. 4D); and the same results were obtained when RNF166 or RNF166 ΔRING were co-expressed with VISA. Also we found overexpression of RNF166 can inhibit the replication of SeV (measured by mRNA level of SeV P protein), while the RING delete mutant enhanced the proliferation of SeV (Fig. 4E). These data indicated that the RING domain is indispensable for the ability of RNF166 to up-regulate cellular anti-RNA virus activity.

We further performed co-immunoprecipitation to detect which domain of RNF166 is required for interactions with TRAF3 and TRAF6. The results showed that the RING deletion and UIM deletion mutants interacted with TRAF3/6 just like full-length RNF166, while the mutant carrying the RING domain only did not (Fig. 4F), suggesting RNF166 interacts with TRAF3 and TRAF6 *via* its zinc finger domain, and the RING domain is necessary for its positive regulatory function.

### RNF166 enhances RNA virus-induced ubiquitination of TRAF3 and TRAF6

**Figure 5:**
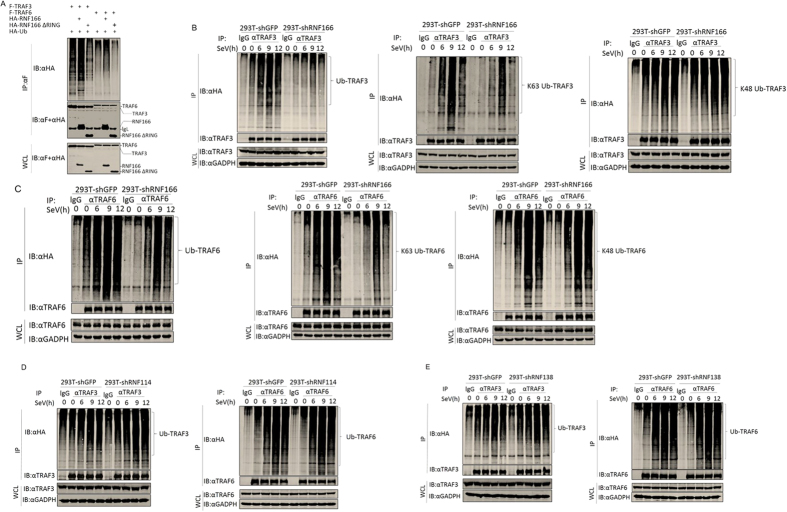
RNF166 enhances virus-mediated ubiquitination of TRAF3/TRAF6. (**A**) Overexpression of RNF166 enhanced the ubiquitination of overexpressed TRAF3/6. 293T cells (1 × 10^7^) were transfected with the indicated plasmids (5 μg each). Cell lysates were immunoprecipitated (IP) with anti-Flag (αF). The immunoprecipitates and whole-cell lysates (WCL) were analyzed by western blot (IB) with anti-HA and anti-Flag antibodies. (**B**,**C**) Knockdown of RNF166 inhibited SeV-mediated WT- and K63- but not K48-linked ubiquitination of endogenous TRAF3 (**B**) and TRAF6 (**C**). Indicated cells (3 × 10^7^) were transfected with WT ubiquitin, K63O ubiquitin, or K48O ubiquitin (5 μg each). At 18 h after transfection, cells were infected with SeV for the indicated times. The lysates were immunoprecipitated with anti-TRAF3 (αTRAF3) or anti-TRAF6 (αTRAF6) antibody. The immunoprecipitates and whole-cell lysates (WCL) were analyzed by western blot (IB) with the indicated antibody. (**D**,**E**) Knockdown of RNF114 (**D**) and RNF138 (**E**) did not inhibit the SeV-mediated ubiquitination of endogenous TRAF3 and TRAF6. Transfection and immunoprecipitation were performed as in (**B**).

It is known that the RING domain is the critical functional domain for E3 ubiquitin ligase, and polyubiquitination of TRAF3 and TRAF6 are important for RLR signaling^13^,^27^, so we suggested that RNF166 could affect the ubiquitination of TRAF3 and TRAF6. We found overexpressed RNF166 enhanced the ubiquitination of TRAF3 and TRAF6 in co-transfection experiments, consistent with previous results, and RNF166 lost this effect when the RING domain was deleted (Fig. 5A).

We then found that the SeV-induced ubiquitination of endogenous TRAF3 and TRAF6 was notably inhibited when RNF166 expression was suppressed (Fig. 5B,C). We further found that K63-linked rather than K48-linked ubiquitination of TRAF3 and TRAF6 was decreased upon SeV infection when RNF166 was knocked down (Fig. 5B,C). However, we did not find an apparent effect of knockdown of endogenous RNF114 and RNF138 on SeV-induced ubiquitination of TRAF3 and TRAF6 (Fig. 5D,E). These data suggested that endogenous RNF166 rather than its homologous proteins RNF114 and RNF138 specifically enhances the SeV-induced ubiquitination of TRAF3 and TRAF6.

## Discussion

Virus-triggered production of IFN-β is critical for the antiviral immune response and is delicately regulated in space and time by various molecules and distinct mechanisms. Ubiquitination has emerged as a critical role in forming signaling complexes and transducing signals downstream^13^.

RNF125 has been reported to negatively regulate RIG-I-mediated antiviral activity *via* conjugating ubiquitin chains to RIG-I and MDA5, leading to their degradation by the proteasome. However, the functions of its homologous proteins RNF114, RNF125, and RNF138 in innate immune signaling pathways remain elusive. Here, we report that RNF166 rather than RNF114, RNF138, and RNF125 potentiates the RNA virus-induced production of IFN-β. Several lines of evidence support this finding. First, overexpressed RNF166 potentiated the SeV- and VISA-mediated induction of IFN-β. Second, knockdown of endogenous RNF166 by shRNA reduced the RNA virus- and dsRNA analog-induced production of IFN-β. Third, RNF166 interacted with endogenous TRAF3 and TRAF6, and these interactions were enhanced upon viral infection. Furthermore, we found that the potentiation of the antiviral effect of RNF166 was mediated by enhancing the virus-induced ubiquitination of TRAF3 and TRAF6. Notably, endogenous RNF114 and RNF138 had no apparent effect on the virus-induced ubiquitination of TRAF3 and TRAF6, and this finding may explain why RNF114 and RNF138 do not enhance SeV-induced antiviral activity while RNF166 did.

TRAF3 and TRAF6 play a vital role in the RIG-I-mediated signaling pathway^29^,^30^,^31^. Therefore, both molecules are functionally regulated by cellular components to control the immune response and viral proteins to evade the host immune system. These regulatory mechanisms include interfering with the interaction between VISA and TRAF3/6^32^,^33^,^34^, deubiquitinating TRAF3/6^26^,^32^,^33^,^34^,^35^, and disrupting the binding of downstream molecules to TRAF3/6^36^,^37^,^38^,^39^. Although a recent study has shown that TRAF3 is dispensable for the RNA virus-induced activation of TBK1 and IRF3^13^, our data suggest that TRAF3 is critical for the SeV-elicited production of IFN-β in HEK293T cells. We cannot exclude the possibility that the differences in the cell lines and experimental methods we used explains the different conclusions. However, we agree that TRAF6 is a critical component downstream of VISA^13^.

In light of our study, RNF166 plays an important role in RNA virus-triggered IFN-β production by enhancing the ubiquitination of TRAF3 and TRAF6. We noted that expression of a mutant with the RING domain deleted, RNF166-∆RING, markedly suppressed VISA-mediated activation of the IFN-β promoter and anti-NDV activity. These data indicate that, as a dominant-negative mutant, RNF166∆RING competes with endogenous RNF166 to bind with TRAF3 or TRAF6, so they cannot be efficiently ubiquitinated and blocks VISA signaling. However, STING has no apparent TRAF-binding motifs that are critical for recruiting TRAFs and can directly recruit IRF3 and TBK1 for activation *via* its carboxyl terminal region^40^; this may explain why RNF166 does not potentiate the cGAS- and STING-induced activation of the IFN-β promoter.

RNF114 was first identified as a psoriasis-susceptibility gene^41^,^42^. Later study revealed that overexpressed RNF114 enhances NF-ΚB and IRF3 reporter activity and increases type I IFN mRNA levels^43^. However, the analysis of cells with RNF114 knockdown yielded a heterogeneous set of results that may have been due to the presence of different populations within the polyclonal cell lines or a redundant role of RNF114^43^. Recently, another independent study revealed that RNF114 acts as a negative regulator of NF-κB-dependent transcription by stabilizing the A20 protein and IκBα^44^. Our data from overexpression and knockdown experiments indicated that RNF114 acts as a negative regulator of SeV-induced IFN-β signaling. A20 has been demonstrated to negatively regulate RIG-I-mediated signaling in several independent studies^45^,^46^,^47^, so it is possible that RNF114 depends on A20 to play negative regulatory roles. However, further investigations are needed to confirm the function of RNF114 in the innate immunity signaling pathway.

Although RNF114, RNF125, RNF138, and RNF166 have similar domain structures, their functions in SeV-induced signaling are quite diverse, including both positive and negative effects. Future investigations into their structures are expected to elucidate the molecular mechanisms underlying their different functions in the innate immunity signaling pathway.

## Methods

### Reagents and cell lines

Mouse antibodies against Flag and HA epitopes (Sigma-Aldrich), rabbit IgG (Sigma-Aldrich), IRDye800-conjugated anti-mouse and anti-rabbit IgG (Rockland Immunochemicals), rabbit polyclonal antibody against TRAF3 (Proteintech), rabbit polyclonal antibody against TRAF6 (Santa Cruz Biotechnology), mouse monoclonal antibody against GADPH (Abmart), poly(I:C) (Pharmacia), mouse polyclonal antibody against VISA (Hong-Bing Shu, Wuhan University, China), NDV-enhanced eGFP (Cheng Wang, Institute of Biochemistry and Cell Biology, Chinese Academy of Sciences, Shanghai, China), Human Influenza A virus (Xin Ye, Institute of Microbiology, Chinese Academy of Sciences, Beijing, China), EMCV (Xin Guo, China Agriculture University, Beijing, China), and HT1080 stably-transfected with ISRE luciferase construct (2fTGH) (Zheng-Fan Jiang, Peking University, China) were from the indicated sources. HEK293T, HeLa, and 2fTGH cells were grown in Dulbecco’s modified Eagle’s medium (DMEM) supplemented with 10% fetal bovine serum (Gibco, Life Technologies) and 100-units/ml penicillin/streptomycin.

### Constructs

Mammalian expression plasmids for Flag- or HA-tagged RNF114, RNF125, RNF138, RNF166, and RNF166 truncations were constructed using standard molecular biology techniques. Mammalian expression plasmids for Flag-RIG-I, -MDA5, -VISA, -TRAF3, -TRAF6, -TBK1, -IRF3, HA-ubiquitin, -K48O ubiquitin, -K63O ubiquitin (Lys-48- and Lys-63-only ubiquitin mutants; all lysine residues except Lys-48 and Lys-63 were mutated to arginine), and IFN-β promoter luciferase reporter plasmids were provided by Dr. Hong-Bing Shu (Wuhan University, Wuhan, China).

### Transfection and Luciferase Assay

HEK293T cells were seeded onto 24-well dishes and transfected the next day with polyethylenimine (Electron Microscopy Sciences). To normalize for transfection efficiency, 50 ng of pRL-Tk *Renilla* luciferase reporter plasmid was added to each transfection. About 18 h after transfection, assays were performed using a dual-specific luciferase assay kit (Promega). Firefly luciferase activity was normalized based on *Renilla* luciferase activity. All reporter assays were repeated at least three times.

### Co-immunoprecipitation and western blot analysis

For transient transfection and immunoprecipitation experiments, HEK293T cells (2 × 10^6^) were transfected with the indicated plasmids for 20 h. The transfected cells were lysed in 0.5 ml lysis buffer (20 mM Tris [pH 7.5], 150 mM NaCl, 1% Triton X-100, 1 mM EDTA, 10 mg/ml aprotinin, 10 mg/ml leupeptin, and 1 mM phenylmethylsulfonyl fluoride). For each immunoprecipitation, a 0.4-ml aliquot of lysate was incubated with 0.5 μg of the indicated antibody and 25 μl of a 1:1 slurry of protein A-Sepharose (GE Healthcare) for 4 h. The Sepharose beads were washed three times with 1 ml lysis buffer. The precipitates were analyzed by western blotting with the indicated antibodies and visualized by incubation with IRDye800-conjugated secondary antibodies using an Odyssey infrared imaging system (Licor Inc.).

### Immunofluorescent staining

Cells were fixed in ice-cold methanol for 20 min at −20 °C, rehydrated three times with phosphate-buffered saline (PBS), and blocked in 5% bovine serum albumin–PBS for 30 min. The cells were stained with primary antibody in blocking buffer for 1 h at 37 °C, rinsed in PBS, and stained again with Alexa Fluor 488-labeled rabbit anti-mouse IgG or Alexa Fluor 594-labeled goat anti-rabbit IgG (Life Technologies) for 1 h at 37 °C. The cells were then rinsed with PBS containing 4′, 6-diamidino-2-phenylindole (DAPI) and mounted. The cells were observed under a confocal microscope (LSM 710 NLO and DuoScan System, Zeiss).

### Q-PCR

mRNA was isolated from cells using TRIzol reagent (Tiangen Biotech) and reverse-transcribed into cDNA with reverse transcriptase (Fermentas). cDNA was amplified on an ABI7300 Detection System (Applied Biosystems) using SYBR Green PCR Master Mix (Takara) according to the manufacturer’s instructions, and data were normalized by the level of β-actin in each individual sample. The 2^−ΔΔCt^ method was used to calculate relative expression changes. The gene-specific primers for Q-PCR were: IFN-β: 5′-AGGACAGGATGAACTTTGAC-3′ and 5′-TGATAGACATTAGCCAGGAG-3′; β-actin: 5′-ACGTGGACATCCGCAAAGAC-3′ and 5′-CAAGAAAGGGTGTAACGCAACTA-3′; RNF114: 5′-CGAGAGCACAGAGACTTC-3′ and 5′-AGGACAGTAAGGACAAGGA-3′; RNF125: 5′-CGTTCCTGTATTGCTACCA-3′ and 5′-CTTCTGACAAGTCCGAATATG-3′; RNF138: 5′-GGAACAGCAATAGGAGTGA-3′ and 5′-CTGGTAATCTGGCTAGGATC-3′; RNF166: 5′-AAGGTGACCCTGGCAAAGAT-3′ and 5′-GGGATAGGCTGTGATGTGGG-3′; ISG15: 5′-AGGCAGCGAACTCATCTTTG-3′ and 5′-CCAGCATCTTCACCGTCAG-3′; MX1: 5′-GTTTACCAGACTCCGACACGA-3′ and 5′- TTCCAGTGCCTTGATTTGCT -3′; P protein of SeV: 5′-TGTTATCGGATTCCTCGACGCAGTC-3′ and 5′-TACTCTCCTCACCTGATCGATTATC-3′.

### IFN-β Bio-assay

The supernatants collected from HEK293T and HeLa cells were added to 2fTGH cells (stably transfected with the ISRE luciferase construct). After 5 h incubation, 2fTGH cells were lysed, and firefly luciferase activity was measured using a luciferase assay kit (Promega).

### Generation of stable knockdown cell pools

Plasmids encoding lentiviruses expressing short hairpin RNAs (shRNAs) were obtained from the library of the RNAi Consortium (Sigma-Aldrich). shRNF166-#1 (TRCN0000007800), #2 (TRCN0000007801), #3 (TRCN0000007802), #4 (TRCN0000007803), #5 (TRCN0000007804); shRNF114 (TRCN0000004485); shRNF125 (TRCN0000004234); shRNF138-#1 (TRCN0000033835), shRNF138-#2 (TRCN0000033837); shTRAF3 (TRCN0000034222); shTRAF6 (TRCN0000007352); and shVISA (TRCN0000148945) plasmids were purified and then transfected into HEK293T cells with a three-plasmid system to produce lentivirus. HEK293T and HeLa cells were plated onto 6-well plates at ~5 × 10^5^ cells per well with 2 ml complete medium. One milliliter of the indicated lentivirus was added with 8 μg/ml polybrene. The plates were incubated for 48 h, and the cells were selected with 2 μg/ml puromycin. Cells were collected for analysis 72 h after selection.

### Generation of TRAF3/6 knockout cell lines

TRAF3 or TRAF6 knockout in HEK293T cells was generated by the CRISPR/CAS9 system containing pCDNA3.1-Cas9 and gRNA (a gift from Dr. Jian-Zhong Xi, Peking University, China) which has been described^48^. Five pairs of gRNA to target TRAF3 or TRAF6 were designed and transfected with Cas9 into HEK293T cells. Twenty-four hours after transfection, the genomic DNAs were extracted for PCR amplification and the indels confirmed by sequencing. The confirmed cell pools were diluted to generate monoclones which were tested by western blot using TRAF3 or TRAF6 antibody to identify gene-knockout cell lines. TRAF3-Cas9 target site: CCAGTTTTTGTCCCTGAACA; TRAF6-Cas9 target site: GTCTGAAAGTGACTGCTGTG. Sequencing primers of TRAF3: 5′-GGATTCTTGTGTTGCCTAA-3′ and 5′-TGGACTTCTTTAGGGCTTT-3′; sequencing primers of TRAF6: 5′-ATTACAACTGAGGTGCTTCT-3′and 5′-GCATCTGGTTCTGTTATAGGA-3′.

### Statistical analysis

Two-way ANOVA analysis were used to analyze data. Experiments were repeated at least three times. Results were considered significant at p < 0.05.

## Additional Information

**How to cite this article**: Chen, H.-W. *et al.* Ring finger protein 166 potentiates RNA virus-induced interferon-β production *via* enhancing the ubiquitination of TRAF3 and TRAF6. *Sci. Rep.* **5**, 14770; doi: 10.1038/srep14770 (2015).
